# Multiple Mutations in Mycobacterium tuberculosis MmpL3 Increase Resistance to MmpL3 Inhibitors

**DOI:** 10.1128/mSphere.00985-20

**Published:** 2020-10-14

**Authors:** Matthew B. McNeil, Theresa O’Malley, Devon Dennison, Catherine D. Shelton, Bjorn Sunde, Tanya Parish

**Affiliations:** a TB Discovery Research, Infectious Disease Research Institute, Seattle, Washington, USA; b Seattle Children’s Research Institute, Seattle, Washington, USA; Antimicrobial Development Specialists, LLC

**Keywords:** cell wall, mycobacteria, antibiotic resistance, drug discovery, mycolic acids, mechanism of resistance, mode of action

## Abstract

The Mycobacterium tuberculosis protein MmpL3 performs an essential role in cell wall synthesis, since it effects the transport of trehalose monomycolates across the inner membrane. Numerous structurally diverse pharmacophores have been identified as inhibitors of MmpL3 largely based on the identification of resistant isolates with mutations in MmpL3. For some compounds, it is possible there are different primary or secondary targets. Here, we have investigated resistance to the spiral amine class of compounds. Isolation and sequencing of resistant mutants demonstrated that all had mutations in MmpL3. We hypothesized that if additional targets of this pharmacophore existed, then successive rounds to generate resistant isolates might reveal mutations in other loci. Since compounds were still active against resistant isolates, albeit with reduced potency, we isolated resistant mutants in this background at higher concentrations. After a second round of isolation with the spiral amine, we found additional mutations in MmpL3. To increase our chance of finding alternative targets, we ran a third round of isolation using a different molecule scaffold (AU1235, an adamantyl urea). Surprisingly, we obtained further mutations in MmpL3. Multiple mutations in MmpL3 increased the level and spectrum of resistance to different pharmacophores but did not incur a fitness cost *in vitro*. These results support the hypothesis that MmpL3 is the primary mechanism of resistance and likely target for these pharmacophores.

**IMPORTANCE** Mycobacterium tuberculosis is a major global human pathogen, and new drugs and new drug targets are urgently required. Cell wall biosynthesis is a major target of current tuberculosis drugs and of new agents under development. Several new classes of molecules appear to have the same target, MmpL3, which is involved in the export and synthesis of the mycobacterial cell wall. However, there is still debate over whether MmpL3 is the primary or only target for these classes. We wanted to confirm the mechanism of resistance for one series. We identified mutations in MmpL3 which led to resistance to the spiral amine series. High-level resistance to these compounds and two other series was conferred by multiple mutations in the same protein (MmpL3). These mutations did not reduce growth rate in culture. These results support the hypothesis that MmpL3 is the primary mechanism of resistance and likely target for these pharmacophores.

## Abstract (precis)

Mycobacterium tuberculosis is a major global human pathogen, and new drugs and new drug targets are urgently required. Cell wall biosynthesis is a major target of current tuberculosis drugs and of new agents under development. Several new classes of molecules appear to have the same target, MmpL3, which is involved in the export and synthesis of the mycobacterial cell wall. However, there is still debate over whether MmpL3 is the primary or only target for these classes. We wanted to confirm the mechanism of resistance for one series. We identified mutations in MmpL3 which led to resistance to the spiral amine series. High-level resistance to these compounds and two other series was conferred by multiple mutations in the same protein (MmpL3). These mutations did not reduce growth rate in culture. These results support the hypothesis that MmpL3 is the primary mechanism of resistance and likely target for these pharmacophores.

## INTRODUCTION

The causative agent of tuberculosis (TB), Mycobacterium tuberculosis, remains a major public health problem, causing approximately 1.5 million deaths in 2019 (1). The emergence and spread of drug-resistant strains against first- and second-line agents highlights the need for new therapeutic agents that have activity against both susceptible and resistant strains of M. tuberculosis.

MmpL3 is essential in M. tuberculosis both *in vitro* and *in vivo* in the mouse model of infection (2, 3). MmpL3 transports trehalose monomycolate (TMM) across the cytoplasmic membrane (4). MmpL3 consists of 12 transmembrane helices and exists as a trimer in M. tuberculosis (3, 5) Numerous pharmacophores have been identified as inhibitors of MmpL3, several of which are efficacious in mouse models of infection (2, 4, 6–11). Resistance against these structurally diverse pharmacophores is due to mutations in MmpL3 and is the basis for their classification as MmpL3 inhibitors. Some MmpL3 inhibitors have synergy with other antitubercular agents such as rifampicin and bedaquiline (2, 12–14).

Recent studies of the tetrahydropyraz[1,5-a]pyrimidine-3-carboxamides (THPPs), originally classified as MmpL3 inhibitors, demonstrated that the enoyl-coenzyme A hydratase (EchA6) is the target of this compound series (15). Mutations in MmpL3 instead impaired the import of THPP into M. tuberculosis (15). This result suggests that pharmacophores originally classified as MmpL3 inhibitors may have other bacterial targets. As resistance mutations in MmpL3 can incur a fitness cost in Mycobacterium smegmatis (16, 17), we hypothesized that if MmpL3 pharmacophores have other bacterial targets, successive rounds of resistant mutant isolation should produce resistance mutations in other loci, rather than selecting for the accumulation of multiple mutations in MmpL3. Instead, here we demonstrate that successive rounds of resistant mutant isolation results in the accumulation of mutations in M. tuberculosis MmpL3. Multiple mutations in MmpL3 increased the level and spectrum of resistance to different MmpL3 pharmacophores and did not incur a fitness cost in M. tuberculosis *in vitro*. The results of this study support the hypothesis that MmpL3 is the primary mechanism of resistance against the studied pharmacophores.

## RESULTS

**FIG 1:**
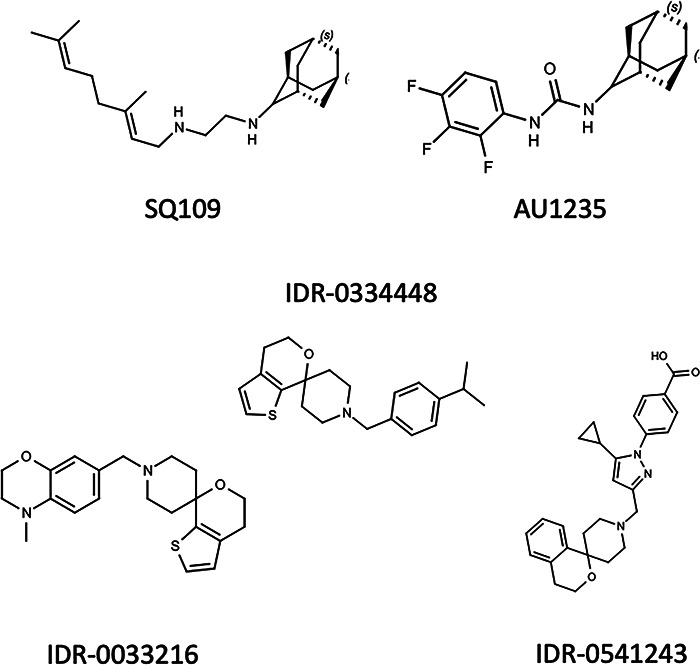
Structures of compounds.

We identified a compound series from a high-throughput screen with activity against M. tuberculosis (Fig. 1) (18). As the compound is part of ongoing drug discovery efforts, we were interested in determining the target of the series to aid in compound progression (our unpublished data). We selected two representative molecules and isolated resistant mutants on solid medium at 5× MIC. We confirmed the resistance by measuring MICs on solid medium. For compound IDR-0033216, we obtained isolates with high-level resistance, with an MIC of 6.3 to 100 μM compared to 0.4 μM in the wild type (Table 1). Similarly, for compound IDR-0334448, we obtained mutants with significant shifts, ranging from 1.6 to 12.5 μM (at least a 4-fold shift) (Table 1). The MICs for rifampicin were not significantly changed from that of the wild-type strain (<4-fold difference). Since similar compounds had been identified which targeted MmpL3 (19), we sequenced this gene from 13 independent isolates. All of the resistant isolates had mutations in *mmpL3*; there were five unique mutations (Table 1).

**TABLE 1:** Isolation of M. tuberculosis strains resistant to spiral amines^*a*^

Strain name^*b*^	MmpL3 SNP^*c*^	MIC (μM)		
		Solid medium^*d*^		Rifampicin^*e*^
		IDR-0033216	IDR-0334448	
Wild type		0.4	0.4	0.0045
LP-0033216-RM1	Y252C	100	NT	0.0082
LP-0033216-RM2	F255L	100	12.5	0.016
LP-0033216-RM5	Y252S	100	NT	0.0096
LP-0033216-RM6	G596R	6.3	NT	0.0059
LP-0033216-RM7	Y252S	100	NT	0.0066
LP-0334448-RM1	F255L	50	6.3	0.0086
LP-0334448-RM5	Y252C	50	3.1	NT

a Resistant mutants were isolated on plates containing 5× MIC and confirmed by streaking onto plates with 5× MIC.

b Strains LP-0033216-RM1 to -RM7 were isolated using compound IDR-0033216; strains LP-0334448-RM1 and -RM5 were isolated using compound IDR-0334448.

c SNP, single nucleotide polymorphism.

d MICs were determined on solid medium against either IDR-0033216 or IDR-0334448. NT, not tested.

e MICs for rifampicin were determined in liquid medium. For comparison, the MIC for rifampicin on solid medium against the wild-type strain was 0.013 μM.

For compound IDR-0033216, we saw mutations in three amino acids. Mutations in F255 and Y252 gave rise to high-level resistance (100 μM); the G596R mutation gave rise to resistance at a lower level, although still a 15-fold shift. For compound IDR-0334448, similar mutations were found in F255 and Y252, giving rise to resistant isolates with MICs of 3.1 to 6.3 μM. This was a smaller shift toward resistance of 8- to 15-fold. Since the mutations were similar for both compounds, we tested for cross-resistance and confirmed that the LP-0334448-RM strains were indeed resistant to compound IDR-0033216 to a high level (Table 1). Thus, mutations in MmpL3 are clearly linked to resistance for this series. It is possible that resistance was conferred by mutations occurring elsewhere in the genome and that MmpL3 mutations were coincidental, but given the number of isolates all with mutations in the same gene, this is unlikely.

### Isolation of mutants with increased resistance.

MmpL3 is essential in M. tuberculosis and Mycobacterium smegmatis (2, 20), and resistance mutations in MmpL3 can incur a fitness cost in M. smegmatis (4, 16). We hypothesized that if additional targets for the series existed, then successive rounds of resistant mutant isolation in strains harboring MmpL3 mutations could identify mutations in those loci. Since the strains were all highly resistant to IDR-0033216, we used IDR-0334448 in the second round. We used strain LP-003216-RM2 with MmpL3_F255L_ as the parental strain, since this mutation is associated with resistance to multiple pharmacophores (Table 1). We isolated mutants with increased resistance by plating onto solid medium with 5× MIC of the resistant isolate (MIC = 12.5 μM). The frequency of resistance was 1.6 × 10^−8^, which is comparable to that for the wild-type (WT) strain. We confirmed 12 strains with a >2-fold shift in MIC compared to that for the parental strains (Table 2). We hypothesized that if additional targets were mutated, then these resistant mutants would not contain additional mutations in MmpL3. Instead, sequencing of MmpL3 in each strain demonstrated that they all contained a second mutation in MmpL3. Five unique mutations were identified (M723T, L567P, M649T, V646M, and V285A) (Table 2).

**TABLE 2:** Sequential isolation of M. tuberculosis resistant isolates using a spiral amine^*a*^

Strain name	MmpL3 SNP(s)	MIC (μM) on solid medium		
		IDR-0334448	SQ109	AU1235
H37Rv-LP	WT	0.4	1.6	0.4
LP-0033216-RM2	F255L	12.5	6.4	0.2
LP-0334448-RM102	F255L, L567P	50	26	0.4
LP-0334448-RM107	F255L, V646M	50	13	0.4
LP-0334448-RM103	F255L, M649T	50	13	0.8
LP-0334448-RM101	F255L, M723T	50	3.2	0.8
LP-0033448-RM113	F255L, V285A	50	13	0.4

a Resistant mutants were isolated by plating the strain LP-0033216-RM2 onto plates containing 5× MIC of IDR-0334448 and confirmed by streaking onto plates with 5× MIC. Strains LP-0334448-RM102 to-RM113, containing five unique mutations, are shown.

We tested for cross-resistance against two other well-studied MmpL3 inhibitors (SQ109 and AU1235) (21, 22). Strains carrying alleles with V285A, L567P, V646M, and M649T were cross resistant to SQ109, whereas none of the strains showed a significant change in susceptibility to AU1235.

### Additional mutations in MmpL3 increase the spectrum of resistance.

In our previous two rounds of resistant mutant isolation, we did not find any resistant strains with mutations outside MmpL3. Since the strains with two mutations now had relatively high-level resistance to both of our original compounds (>50 μM) (Tables 1 and 2), we were unable to attempt further rounds of resistant strain isolation to IDR-0334448 or IDR-0033216. However, the spectrum of cross-resistance to SQ109 and AU1235 was variable (Table 2), as might be expected if compounds have unique interactions with MmpL3 (23). Therefore, we made use of the observation that the strains were not fully resistant to these compounds and conducted a third round of resistant mutant isolation. We selected two strains, which demonstrated different levels of resistance to SQ109 and no significant resistance to AU1235: (i) LP-0334448-RM102 with F255L and L567P showed a 5.5-fold resistance to SQ109, and (ii) LP-0334448-RM107 with F255L and V646M showed a 3.3-fold increase in resistance to SQ109. These mutations were chosen as they provide resistance against other pharmacophores and are functionally important residues (5, 24, 25). Since the MICs of AU1235 were <2-fold changed, we were able to use this compound to isolate resistant mutants at 5× MIC on solid medium as before.

Again, we hypothesized that if additional targets were mutated, then these isolated resistant mutants would not contain mutations in MmpL3. Instead, sequencing of nine strains (three from the LP-0334448-RM102 strain and six from the LP-0334448-RM107 strain) demonstrated that all had additional mutations at F644, either F644L or F644I (Table 3) and had high-level resistance to AU1235 (16-fold and 100-fold shifts, respectively). These strains were also cross resistant to SQ109, albeit at a lower level (5-fold and 6.7-fold, respectively). F644L did not result in increased resistance to a structurally related spiral amine (i.e., IDR-0541243), while F644I conferred a 7-fold shift to resistance (Table 3). This is consistent with the predicted functional importance of F644 and the targeting of this site by multiple pharmacophores (5).

**TABLE 3:** Sequential isolation of M. tuberculosis resistant isolates using AU1235^*a*^

Strain name^*b*^	MmpL3 SNPs	MIC in liquid medium (μM)		
		IDR-0541243	SQ109	AU1235
H37Rv-LP	WT	0.7	0.9	0.3
LP-0334448-RM102	F255L, L567P	6	5	0.7
LP-0497754-RM201	F255L, L567P, F644L	5	25	11
LP-0334448-RM107	F255L, V646M	6	3	0.3
LP-0497754-RM302	F255L, V646M, F644I	40	20	30

a Resistant mutants were isolated on plates containing 5× MIC AU1235 and confirmed by streaking onto plates with 5× MIC.

b Strain LP-0497754-RM201 was derived from strain LP-0334448-RM102; strain LP-0497754-RM302 was derived from strain LP-0334448-RM107.

### Mutations in MmpL3 do not affect *in vitro* fitness.

**FIG 2:**
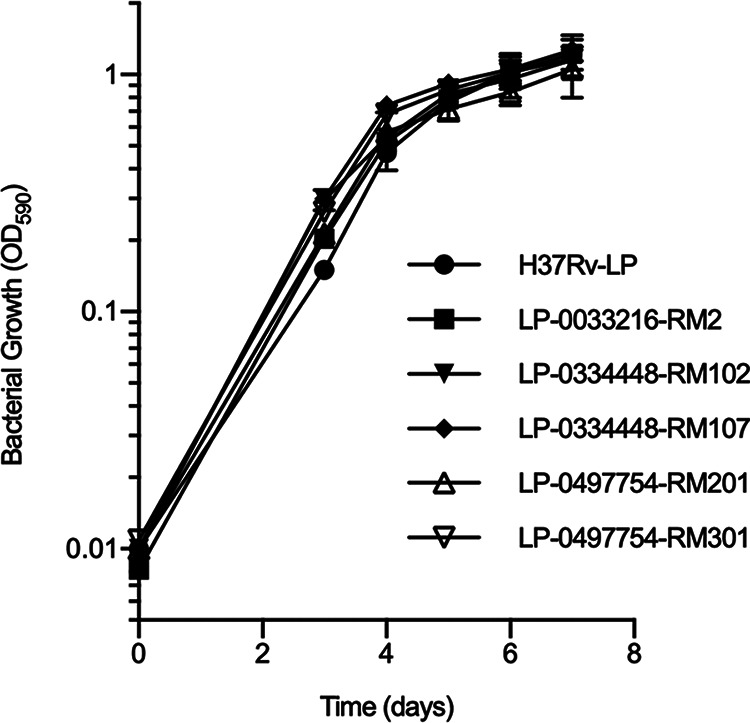
Growth of M. tuberculosis strains carrying mutant MmpL3 alleles. M. tuberculosis strains carrying mutations in MmpL3 were grown in 7H9-OADC-Tw. Results are the means ± SDs from three biological replicates. H37Rv-LP, wild type; LP-0033216-RM2, F255L; LP-0334448-RM102, F255L and L567P; LP-0334448-RM107, F255L and V646M; LP-0497754-RM201, F255L, L567P, and F644L; LP-0497754-RM301, F255L, V646M, and F644I.

Mutations in M. smegmatis MmpL3 have been shown to incur a fitness cost with lower growth rates (16, 17). Since MmpL3 is essential for growth and the mutations occur in key regions, we predicted that some of the M. tuberculosis mutant strains would be similarly impaired. We performed growth curves of representative single, double, and triple MmpL3 mutants in standard Middlebrook 7H9-oleic acid-albumin-dextrose-catalase (OADC)-Tween medium. However, none of strains demonstrated any growth impairment compared to WT H37Rv (Fig. 2). Thus, the accumulation of resistance mutations in MmpL3 is not associated with an *in vitro* growth defect in M. tuberculosis. None of the mutant strains showed abnormal colony morphology.

### Resistance residues map to the transmembrane segments of MmpL3.

MmpL3 consists of 12 transmembrane segments, two large periplasmic domains, and a nonessential C-terminal cytoplasmic domain (5). The majority of previously identified resistance mutations in MmpL3 map to the middle of transmembrane helices that, upon protein folding, are located in close proximity to the interior vestibule region that forms a predicted proton relay. Consistent with previous findings (5, 26), the majority of resistance residues in this study map to the transmembrane segments of MmpL3. One residue (M649) that does not map to a transmembrane segment is located in small cytoplasmic loops in close proximity to the inner membrane.

## DISCUSSION

The identification of EchA6 as the biological target of THPPs has raised concerns as to whether MmpL3 is the biological target of other diverse pharmacophores (15). We hypothesized that if additional targets of the spiral amine pharmacophore existed, then they may be identified by successive rounds of resistant mutant isolation. Instead, we observed the accumulation of mutations in MmpL3. Multiple mutations in MmpL3 generally resulted in increased resistance to multiple compound classes. This accumulation of multiple mutations resulting in increased levels and spectrum of resistance is analogous to the accumulation of mutations in *gyrA* and *gyrB* and their associated resistance to fluoroquinolones (27–29). These data support previous conclusions that MmpL3 is the primary mechanism of resistance against these diverse pharmacophores.

We identified several residues within the transmembrane segments of MmpL3 that are associated with resistance to the spiral amine class (Y252, F255, V285, L567, G596, F644, V646, M649, and M723). Some of these mutations, such as F255L and F644I/L, have been observed to confer resistance to other pharmacophores (4, 8–11, 14, 24, 30). Consistent with previous genetic and biochemical studies, these data suggest that these structurally diverse pharmacophores bind to overlapping regions within the transmembrane domains of MmpL3 (17, 23). Furthermore, this study demonstrates that combining multiple mutations increases both the level and spectrum of resistance against diverse pharmacophores. Combined, these results further strengthen MmpL3 as a promising drug target in M. tuberculosis that is inhibited by a number of structurally diverse pharmacophores.

## MATERIALS AND METHODS

### Bacterial culture.

M. tuberculosis H37Rv (ATCC 25618) was grown in Middlebrook 7H9 medium containing 10% oleic acid-albumin-dextrose-catalase (OADC) supplement (Becton, Dickinson) and 0.05% (wt/vol) Tween 80 (7H9-OADC-Tw) at 37°C. M. tuberculosis was grown in either 100 ml of medium in a 450-cm^2^ roller bottle at 100 rpm or in 5 ml of medium in a 16-mm borosilicate tube containing an 8-mm stir bar with stirring at 250 rpm. Solid medium was Middlebrook 7H10 agar plus 10% (vol/vol) OADC.

### Determination of MIC.

MICs were determined on either solid or liquid media as described (31, 32). For solid medium, MICs were determined in 24-well plates inoculated with 5 μl of culture at 1 × 10^5^ CFU/ml. Plates were incubated at 37°C for 4 weeks, and the MIC was defined as the minimum concentration that prevented growth. For liquid medium, assays were performed in 96-well plates inoculated with 35 μl of culture at an optical density at 590 nm (OD_590_) of 0.06 to 0.10; growth was measured by OD_590_ after 5 days at 37°C. The MIC_90_ (IC_90_) was defined as the concentration at which 90% of growth was inhibited. All compounds were dissolved in dimethyl sulfoxide (DMSO). SQ109 was purchased through Sigma (SML1309). AU1235 was synthesized according to published protocols (4). MIC determinations were performed in at least biological duplicates.

### Isolation of resistant mutants.

Resistant mutants were isolated by plating M. tuberculosis (OD_590_ of ∼0.8) on 7H10-OADC agar containing 5× MIC for each compound as previously described (8). Plates were incubated at 37°C until colonies appeared (3 to 6 weeks). Colonies were streaked onto 7H10-OADC agar plates containing 5× MIC to confirm resistance.

### Sequencing.

Genomic DNA was extracted using a chloroform-methanol inactivation followed by a phenol-chloroform precipitation of DNA using previously published protocols (8). Mutations were identified by amplifying *mmpL3* (*rv0206c*) from extracted genomic DNA (gDNA) using the primer pairs MmpL3F1-JJ01 (5′-GCT GTT GAC CTC GCG AGT GTG-3′) and MmpL3R1-JJ01 (5′-GCT TTC TTC AAC AAT GCG GTG CAG-3′). Sequencing was carried out using primers MmpL3F1-JJ01, MmpL3R1-JJ01, MmpL3_SMF2.5 (5′-TAT CCC GGT GGG CAA GCT GTC-3′), MmpL3F2-JJ01 (5′-CAA CGG CGA ATG GAA GTG CTG-3′) and MmpL3F3s-JJ01 (5′-CGC CCT GGA GCT GGA TTC AAT C-3′). Sanger sequencing was performed through GENEWIZ, with resulting sequencing reads mapped to H37Rv *mmpL3* (*rv0206c*) using SeqMan-Pro (DNASTAR-Lasergene) with mutations identified.
